# Predictive modelling and optimization of WEDM of nickel aluminium bronze alloy using optimised support vector regression and evolutionary algorithm

**DOI:** 10.1038/s41598-025-34151-8

**Published:** 2025-12-30

**Authors:** Subhankar Saha, Sri Srinivasa Raju Modampuri, Hrishikesh Dutta, Rammohan Mallipeddi, Dhanaraj Savary Nasan, Mridusmita Roy Choudhury

**Affiliations:** 1 Department of Mechanical Engineering, Brainware University, Kolkata, India; 2 Center for Multidisciplinary Research and Innovations, Brainware University, Kolkata, India; 3 College of Computer Science, Sichuan University, Chengdu, 610065 China; 4 Centre for Additive Manufacturing, Chennai Institute of Technology, Chennai, Tamil Nadu 600069 India; 5 Department of Artificial Intelligence, School of Electronics, Kyungpook National University, Daegu, Republic of Korea; 6 The Institution of Engineers (India), Kolkata, India; 7 Department of Mechanical and Industrial Engineering, Manipal Institute of Technology Bengaluru, Manipal Academy of Higher Education, Manipal, Karnataka 576104 India

**Keywords:** WEDM, NAB alloy, Optimization, Machine learning, OSVR, IBEA-AOG, Engineering, Materials science, Mathematics and computing

## Abstract

The primary objective of this study was to develop an accurate predictive framework and an efficient multi-objective optimisation strategy for wire electric discharge machining (WEDM) of NAB alloy, focusing on Cutting Speed (CS) and Surface Roughness (SR). An optimized Support Vector Regression (OSVR) model was constructed to capture the complex and stochastic input–output relationships inherent to the spark erosion process. The model exhibited excellent predictive accuracy, with MSE = 0.0027 and R^2^ = 0.9970 for CS and MSE = 0.0012 and R^2^ = 0.9924 for SR, validated through scatter and stem plots. To optimise the conflicting objectives of maximising CS and minimising SR, an adaptive offspring generation-driven indicator-based evolutionary algorithm (IBEA-AOG) was applied. The algorithm generated 100 Pareto-optimal solutions and outperformed twelve state-of-the-art algorithms, as confirmed by Friedman mean rank and Nemenyi tests. Spearman correlation analysis was used to assess the influence of process parameters on CS and SR. Surface integrity was further evaluated using field emission scanning electron microscopy (FESEM) and non-contact optical profilometry. High discharge energy settings produced surfaces with globules, large melted deposits, and overlapping craters due to poor dielectric flushing, while low discharge energy led to smoother surfaces with fewer craters and thinner recast layers. Microcracks were observed under all conditions, but were more pronounced at higher energies owing to steeper thermal gradients. Overall, the OSVR-IBEA-AOG hybrid framework proved highly effective for prediction, analysis, and multi-objective optimisation in WEDM.

**Supplementary Information:**

The online version contains supplementary material available at 10.1038/s41598-025-34151-8.

## Introduction

Wire electric discharge machining (WEDM) is a spark erosion process that performs machining through spark discharges^1^. As a result, several physical phenomena, including wire motion and table motion, the circulation of dielectric fluid, the discharge variant, the energy content of spark discharge, and the discharge frequency of spark, must be considered when modelling the process. This complexity renders the process extremely stochastic. Employing numerical and analytical methods to model the WEDM process enhances understanding of the underlying physics; however, it necessitates a considerable amount of time and extensive computations, which may not be advantageous from a smart manufacturing perspective. In contrast, machine learning models, developed by analysing data obtained from experiments, are significantly more effective at elucidating the WEDM process. Consequently, several research groups have deployed machine learning approaches to unleash potential insights about the WEDM process^2–4^. In a research investigation it is found that artificial neural network (ANN) performs better than dimensional analysis approach in prediction of WEDM performances for Al/SiCp MMC, considering input features such as the thermal conductivity of the workpiece, material, wire feed rate, pulse on time, coefficient of thermal expansion, pulse off time, density, and wire tension^5^. Singh et al.^6^ predicted surface roughness using an efficient support vector regression (SVR) technique, with results closely matching the experimental responses for WEDM of AA6063. Recently, Nain et al.^7^ predicted the material erosion rate and surface roughness of machined surfaces by leveraging support vector regression models with various kernel variants. They further conducted a sensitivity analysis using the best eligible model to comprehend the relative importance of process variables. A group of researchers attempted to combine the GPR model with a metaheuristic algorithm to predict and track the optimal operating conditions for material erosion rate and surface characteristics during WEDM processing of SiCp/Al composite material^8^. Two significant WEDM outcomes have been predicted using the decision tree algorithm and the Naïve Bayes algorithm for WEDM of aluminium composites^9^. Kumar et al.^10^ proposed a hybrid strategy that integrates a desirability function with a machine learning algorithm to optimise process parameters in WEDM of CP-Ti G material. Shukla et al.^11^ applied the gradient descent method to optimise surface roughness and kerf width during WEDM of Hastelloy C-276. Sibalija et al.^12^ utilised a Bayesian regularised neural network combined with evolutionary algorithms to simultaneously optimise multiple responses (i.e., gap current, surface roughness, and cutting speed). A research group employed ANN to forecast acoustic emission (AE) signals and surface roughness in WEDM of titanium^13^. Chou et al.^14^ used an artificial neural network (ANN) to predict wire rupture in a study that included process variables such as gap voltage, water resistance, and feed rate. A Deep Neural Network (DNN) has been proposed to model the wire wear ratio and surface roughness in WEDM of AISI4140 steel^15^. Naresh et al.^16^ determined that the prediction accuracy of ANFIS exceeds that of ANN in the WEDM of Nitinol. Recently, researchers mapped WEDM process responses with explanatory variables using ANN and RSM while working with the A286 superalloy^17^. Abhilash et al.^18^ investigated wire breakage prediction through ANFIS in WEDM of Inconel 718 superalloy, considering mean gap voltage variation as the response and Ton, SV, Toff, and WF as input features. This research group also proposed an offline ANN-based classifier to forecast different conditions, such as wire breakage, spark absence, and normal machining for various parametric combinations^19^. Zhang and his co-researchers suggested a two-stage classification for discharging pulse discrimination using SVM for rough classification, followed by fine classification through the Random Forest algorithm^20^. Abhilash and his team reported that the Naïve Bayes classifier is computationally efficient for classifying wire breakage cases, aiming to improve sustainability in WEDM^21^. Recently, a research team highlighted the significance of selecting clustering algorithms on the predictive prowess of ANFIS when estimating process outputs like material erosion rate and surface roughness. The predictive capability of ANFIS based on the grid-partitioning model is relatively superior to that of the subtractive clustering model^22^. A team of researchers proposed a framework in which synthetic surface images generated by a Singular Generative Adversarial Network (SinGAN) are used in machine learning models to predict the surface morphology of WEDM machined Nitinol specimens^23^. Two researchers introduced a non-dominated sorted genetic algorithm to identify multiple sets of optimal parameters for two performance metrics (i.e., Pareto set) during the WEDM of Ti6Al4V^24^. Similarly, a research team reported a group of Pareto-optimal solutions for conflicting objectives (Cutting Rate and Surface Roughness) in the WEDM of Ti 6-2-4-2 alloy by employing the NSGA II algorithm^25^. Gao et al.^26^ compounded Dung Beetle Optimiser driven BPNN with multi-objective particle swarm optimization to maximize the material removal rate while minimising the kerf width, and surface roughness in WEDM of polysilicon ingots. Sun et al.^27^ investigated the WEDM of Ti-6Al-4 V alloy using a four-factor, three-level full factorial experiment to study the effects of pulse-on time, pulse-off time, peak current, and open voltage on surface roughness, material removal rate, and kerf width. Artificial neural networks were developed to model process behaviour, while NSGA-II was applied for multi-objective optimization and EWM-TOPSIS for solution ranking. The optimal parameters identified were Ton = 8.44 µs, Toff = 5.12 µs, IP = 13.9 A, and OV = 209.44 V, providing a practical approach for efficient parameter selection in aerospace applications. Chanie et al.^28^ developed an Artificial Neural Network (ANN) model with an optimized architecture (4-9-2) to predict the response parameters, namely material removal rate and surface roughness in WEDM of charging handlebar. Subsequently, they employed a multi-objective genetic algorithm to determine the optimal parameter settings: a peak current of 2.513 A, a pulse-on time of 25.642 µs, a wire feed rate of 9.999 m/min, and a pulse-off time of 7.975 µs. Sharma et al.^29^ carried out machine learning (polynomial regression model) assisted single response optimization and multiple response optimization in WEDM of Titanium alloy (Ti-6Al-7Nb). The optimal combination of inputs obtained by simultaneous optimization of MRR and SR is Ton: 114 mu, Toff: 60 mu, IP: 80 A, and SV: 80; and Ton: 114 mu, Toff: 60 mu, IP: 140 A, and SV: 80 V, as per Genetic algorithm and Teaching Learning Based optimization, respectively. Siyoum et al.^4^ applied an Artificial Neural Network (ANN) to model machining time and surface roughness, which were subsequently optimized using the Multi-Objective Jaya Algorithm, Genetic Algorithm, and Teaching–Learning-Based Optimization. The Multi-Objective Jaya Algorithm yielded the best performance, attaining a surface roughness of 193 μm and a machining time of 183.469 s under optimal parameters: pulse-off time 14.291 µs, peak current 2.438 A, pulse-on time 8.391 µs, and wire feed rate 21.274 mm/s.

Although numerous investigations have applied machine learning and optimisation techniques to model and enhance the WEDM process, several important limitations remain unaddressed. First, most existing studies focus on materials such as aluminium composites, titanium alloys, superalloys, and tool steels, whereas research dedicated specifically to the WEDM of nickel aluminium bronze (NAB) alloy is nearly absent, despite its industrial relevance in marine, aerospace, and corrosion-resistant applications. Second, many machine-learning-based predictors—including ANN, SVR, ANFIS, GPR–metaheuristic hybrids, and deep neural architectures—often exhibit restricted generalisation capability due to limited dataset sizes and insufficient hyperparameter tuning. These studies typically predict only single responses or a limited set of outputs and rarely address simultaneous multiobjective optimisation for conflicting performance metrics such as cutting speed (CS) and surface roughness (SR).

Additionally, the majority of optimisation-driven works rely on traditional evolutionary algorithms (e.g., NSGA-II, MOPSO) without incorporating adaptive or indicator-based mechanisms that enhance convergence efficiency and diversity maintenance. In several cases, explainability and variable-importance analyses are insufficient or missing, restricting the interpretability of the models and limiting their applicability in real manufacturing settings. Rigorous statistical validation techniques, such as non-parametric ranking tests, are also underutilised, reducing confidence in the comparative superiority of optimisation methods. Moreover, comprehensive surface morphology validation, which is essential for correlating predicted responses with actual machining outcomes, is sparsely reported across existing literature.

To address these limitations, the present study develops a comprehensive and robust modelling–optimisation framework tailored to the WEDM of NAB alloy. An optimised support vector regression (OSVR) model is employed as a surrogate to capture the nonlinear relationships between key process variables and two critical performance metrics: cutting speed (CS) and surface roughness (SR). The optimised surrogate model is then utilised to predict these responses for unknown combinations of machining parameters, enabling efficient exploration of the solution space.

To simultaneously optimise the two conflicting responses, this work introduces an Indicator-Based Evolutionary Algorithm with Adaptive Offspring Generation (IBEA-AOG). The proposed algorithm is benchmarked against eleven state-of-the-art multiobjective optimisation algorithms, and its effectiveness is rigorously assessed using non-parametric statistical tests, including the Friedman ranking test and the Nemenyi post-hoc test. A detailed parametric study using contour plots and a Spearman correlation-based sensitivity analysis is conducted to reveal the influence and importance of individual process variables. Furthermore, the study incorporates field emission scanning electron microscopy (FESEM) to investigate the surface morphology of the machined NAB alloy, providing physical validation of the predicted trends.

## Materials and methods

**Fig. 1:**
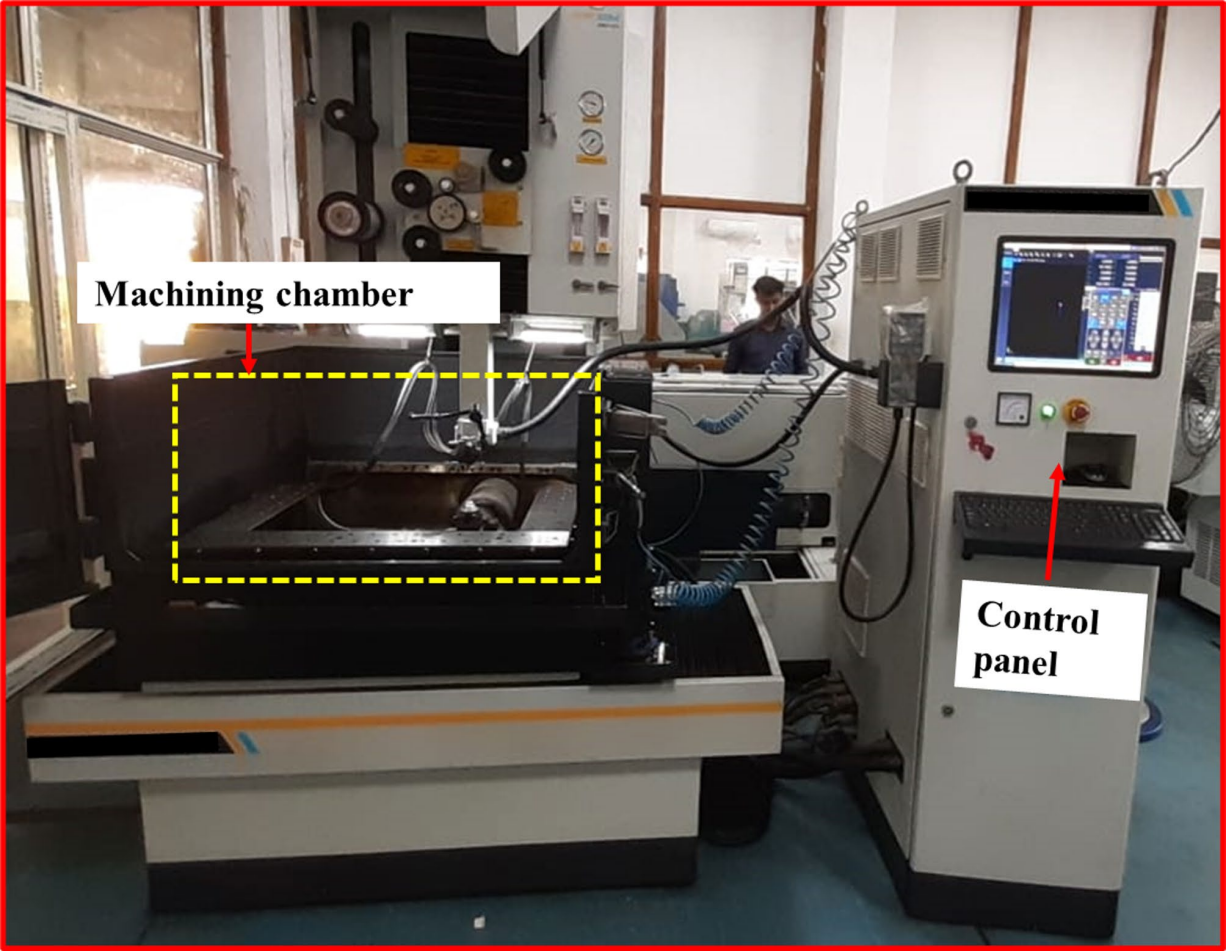
WEDM set up.

**Fig. 2:**
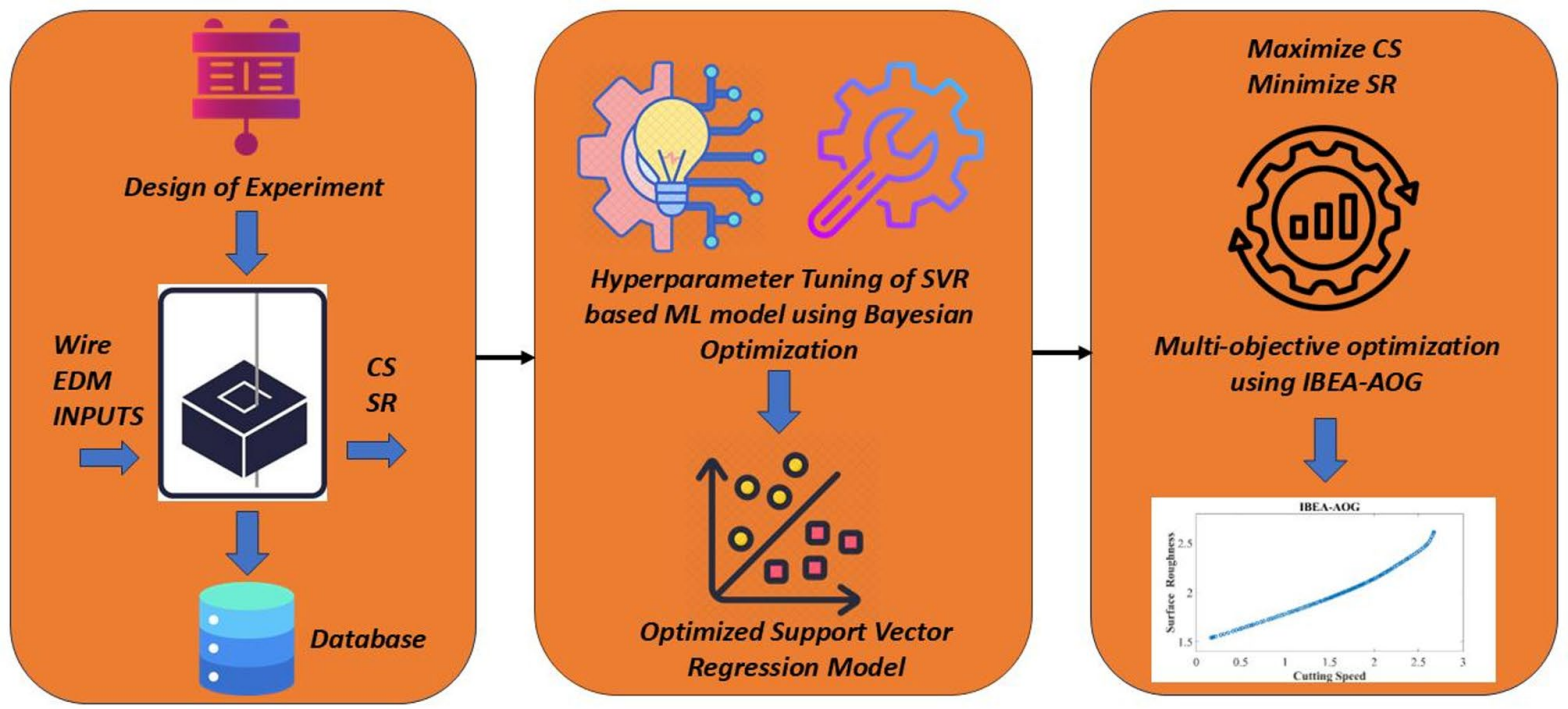
Workflow of the current article.

In the present study, the material used is Nickel Aluminium Bronze (NAB) alloy. Machining experiments were carried out using a WEDM system (Electronica Tool Master 6s) with a table size of 875 × 595 mm and an accuracy of 10 μm (Fig. 1) to generate a complex contour on the NAB alloy. The intricate profile is cut on a rectangular plate having the dimensions of 12 mm × 5 mm × 10 mm. The tool electrode used for cutting the samples is zinc-coated brass wire. Face-centred Central Composite Design (CCD) scheme has been enforced to conduct the 30 experiments with four explanatory variables, i.e., pulse on time (Pon), pulse off time (Poff), peak current (Ip), and servo voltage (SV) and two output variables, i.e., CS, and SR. Pilot experiments and a thorough literature review are used to determine the process variables and their corresponding levels (see Table 1). The workflow that demonstrates the main theme of this article is shown in Fig. 2. To attenuate experimental error, each experiment was performed three times, and the average responses (i.e., CS and SR) were obtained (Table 2). CS is directly retrieved from the monitor, whereas SR, which is measured in terms of Ra, has been retrieved from the surface roughness tester. The influence of process parameter variations on the response variables was examined through contour plots generated using Minitab^®^ statistical software (Version 17, Minitab LLC, USA; https://www.minitab.com). Additionally, surface topographic features of the WEDM surfaces are examined using FESEM.

**Table 1:** Ranges of process parameters.

Process parameters	Levels		
	Level 1	Level 2	Level 3
Pulse on time (Pon)	115	125	135
Pulse off time (Poff)	55	60	65
Peak current (Ip)	12	14	16
Servo voltage (SV)	35	45	55

**Table 2:** Experimental results.

Test number	Pon (μs)	Poff (μs)	Ip (A)	SV (volt)	CS ± Std. (m/min)	SR ± Std. (micron)
1	135	55	14	45	2.38 ± 0.03	2.29 ± 0.014
2	135	60	14	45	2.23 ± 0.045	2.24 ± 0.023
3	115	65	12	55	0.22 ± 0.025	1.86 ± 0.027
4	135	60	14	45	2.23 ± 0.033	2.25 ± 0.035
5	115	55	16	55	1.89 ± 0.035	2.74 ± 0.033
6	135	55	16	55	2.22 ± 0.027	2.63 ± 0.012
7	135	55	16	35	2.37 ± 0.023	2.65 ± 0.017
8	135	65	16	55	2.08 ± 0.033	2.53 ± 0.028
9	135	60	14	45	2.23 ± 0.037	2.24 ± 0.016
10	135	60	14	55	2.27 ± 0.018	2.26 ± 0.032
11	115	65	16	35	2.23 ± 0.028	2.73 ± 0.034
12	115	60	14	45	2.11 ± 0.032	2.47 ± 0.033
13	115	65	16	55	1.82 ± 0.031	2.72 ± 0.032
14	125	60	12	45	0.25 ± 0.017	1.77 ± 0.030
15	115	65	12	35	0.23 ± 0.043	1.89 ± 0.022
16	115	55	12	55	0.24 ± 0.035	1.92 ± 0.019
17	125	60	14	35	2.52 ± 0.033	2.46 ± 0.035
18	125	60	14	45	2.33 ± 0.021	2.46 ± 0.010
19	135	55	12	55	0.28 ± 0.027	1.69 ± 0.027
20	125	60	16	45	2.36 ± 0.032	2.69 ± 0.035
21	135	60	14	45	2.48 ± 0.036	2.24 ± 0.032
22	135	65	12	55	0.23 ± 0.012	1.6 ± 0.037
23	135	65	12	35	0.30 ± 0.017	1.61 ± 0.011
24	125	60	14	45	2.25 ± 0.019	2.47 ± 0.016
25	135	65	16	35	2.00 ± 0.034	2.55 ± 0.022
26	125	65	14	45	2.20 ± 0.03	2.34 ± 0.025
27	115	55	12	35	0.26 ± 0.029	1.99 ± 0.028
28	125	60	14	45	2.23 ± 0.031	2.45 ± 0.030
29	135	55	12	35	0.31 ± 0.034	1.75 ± 0.015
30	115	55	16	35	2.32 ± 0.011	2.76 ± 0.017

### Support vector regression

SVM is created for linear classification and later extended to non-linear classification and regression (SVR). SVR minimises generalisation error by reducing structural risk in a high-dimensional feature space created through various kernel functions. The training dataset for SVR consists of $\left( x \right)_{n = 1}^M$ where *M* denotes the number of samples, and $x \in {R^d}$ has *d* input features as a vector, and $\left( y \right)_{n = 1}^M$ as the corresponding targets. SVR aims to find a function that is as flat as possible while ensuring the highest deviation $\:\epsilon\:$ from $\:{\left(y\right)}_{n=1}^{M}$. The provided equation can be used to represent the function

1 $$\:\widehat{f}\left(x,w\right)=\sum\:_{n=1}^{M}{w}_{n}.{g}_{n}\left(x\right)+b$$

By minimizing ${\left\| w \right\|^2}$, a small value of *w* guarantees maximal flatness. The following is the problem formulation:

2 $$\:\boldsymbol{M}\boldsymbol{i}\boldsymbol{n}\boldsymbol{i}\boldsymbol{m}\boldsymbol{i}\boldsymbol{z}\boldsymbol{e}\frac{1}{2}{\left\| {\rm{w}} \right\|}^{2}$$

3 $$\:\boldsymbol{S}\boldsymbol{u}\boldsymbol{b}\:\boldsymbol{t}\boldsymbol{o}\:\left|{y}_{n}-\widehat{f}({x}_{n},{w}_{n})\right|\le\:\epsilon\:$$

Slack variables $\:{\xi\:}_{n}^{-},{\xi\:}_{n}^{+}$ and constant $\:C$ is included to deal with a few sample points that have more errors than $\:\varepsilon\:$. It thus modifies the optimization problem as

4 $$\:Minimize\frac{1}{2}{\left\| {\rm{w}} \right\|}^{2}+{C}^{\sum\:_{n=1}^{M}{(\xi\:}_{n}^{+}+{\xi\:}_{n}^{-})}$$

5 $$\:\boldsymbol{S}\boldsymbol{u}\boldsymbol{b}\:\boldsymbol{t}\boldsymbol{o}\:\left|{y}_{n}-\widehat{f}({x}_{n},{w}_{n})\right|\le\:\epsilon\:+{|\xi\:}_{n}^{+}|$$

This structural risk minimization framework allows SVR to generalize well, particularly when combined with kernels such as RBF, polynomial, or sigmoid, which map the input data into high-dimensional feature spaces where nonlinear relationships can be captured efficiently.

The effectiveness of SVR and related machine-learning approaches in energy systems, reliability assessment, and intelligent sensing has been documented in several recent studies. For instance, for probabilistic reliability assessment a probabilistic deep learning combined with GMM-HMM frameworks is employed^30^. SVR and other ML models have also shown strong applicability in AI-enabled data acquisition and fitness pressure measurement^31^, fault early-warning methodologies using Bayesian-optimized XGBoost in ultrasonic flowmeters, and interpretable combinatorial learning approaches for shale fracability evaluation^32^. These studies collectively demonstrate the growing importance of SVR-based and machine-learning-based models in nonlinear prediction, uncertainty handling, and high-dimensional data environments, thereby supporting the methodological foundation adopted in the present work.

### Working mechanism of indicator-based evolutionary algorithm with adaptive offspring strategy (IBEA-AOG)

The operation of IBEA-AOG is similar to IBEA except for the offspring-generating strategy. It starts by initialising a stochastic population (Algorithm 1, Line 1). Adaptive Offspring Generation Strategy stochastically employs SBX and DE crossover to create diversified solutions that produce solutions with dual characteristics, i.e., converging solutions with good diversity. In general, the parent solutions are selected from the vicinity to generate offspring in decomposition strategies, which may not be feasible for all cases. Therefore, to have better exploration, despite selecting the parents from the neighborhood$\:\:B,\:the\:$parents are considered from the whole population on some occasions. Literature suggests that SBX crossover fits for certain kinds of problems, whereas DE crossover fits well for other kinds of problems. In practice, the selection of an operator is a difficult task as there is no knowledge base to decide which operator would be suitable for any problem under consideration. Thus, despite selecting a single operator for the entire evolutionary process, the AOG strategy (Algorithm 1, Line 4) assigns both operators, i.e., SBX and DE crossover, equally to generate offspring members. After the generation of the offspring population, the environmental selection strategy will select the best $\:N$ solutions in terms of convergence and diversity (Algorithm 1, Line 6). The binary quality indicator is employed herewith to compare the two solutions’ quality. The fitness value of each solution $\:\left(x\right)$ in the population ($\:Pop)$ is assigned as follows:

6 $$\:F\left(x\right)=\sum\:_{y\in\:\:Pop}-{e}^{-I(y,x)/k}$$

where $\:I\left(x,y\right)=\mathrm{m}\mathrm{a}\mathrm{x}(f\left(x\right)-f(y\left)\right)$ and $\:k>0$ is a parameter that defines the scaling factor that depends on $\:I$ and the problem. The exponential function used in the fitness value amplifies the dominance relation of the compared solutions. The solution with the maximum fitness value will be preferred for further iterations.

**Table:** Algorithm 1: General framework of IBEA-AOG.

Input:	$\:N$	–	Size of the population
	$\:M$	–	Number of objectives
	$\:MaxGen$	–	Termination criteria (maximum number of generations)
Output	$\:Pop$	–	Population
1.	[$\:Pop,B]$	$\:\leftarrow\:$	$\:Initialize(N,M)$
2.	$\:iter$	=	1
3.	While $\:iter\:<\:\:MaxGen$		
4.	$\:Offspring$	$\:\leftarrow\:$	$\:AOG$($\:Pop$,B)
5.	$\:R$	=	$\:Pop\cup\:Offspring$
6.	$\:Pop$	$\:\leftarrow\:$	$\:Selection\left(R\right)$
7.	$\:iter$	=	$\:iter+1$
8.	End		

## Results and discussion

In this section, sensitivity analyses, SVR-based machine learning models, parametric analyses, multi-objective optimization, and surface topographical aspects have been discussed.

### Sensitivity analyses

In the present research, the influencing process parameters for CS and SR are assessed using the Spearman correlation coefficient heat map. The Spearman correlation coefficient evaluates the degree and orientation of association between two ranked variables^33^. It is mathematically represented as:

7 $$\rho =1 - \frac{{6\sum {d_{i}^{2}} }}{{n\left( {{n^2} - 1} \right)}}$$

where *ρ* is the Spearman correlation coefficient, ${d_i}$ refers to the difference between the two ranks of each observation, and *n* refers to the number of observations. The Spearman Correlation Coefficient takes a value ranging from − 1 to + 1, whereby the value of + 1 refers to positive association, the value of -1 refers to negative association, and 0 refers to null association.

**Fig. 3:**
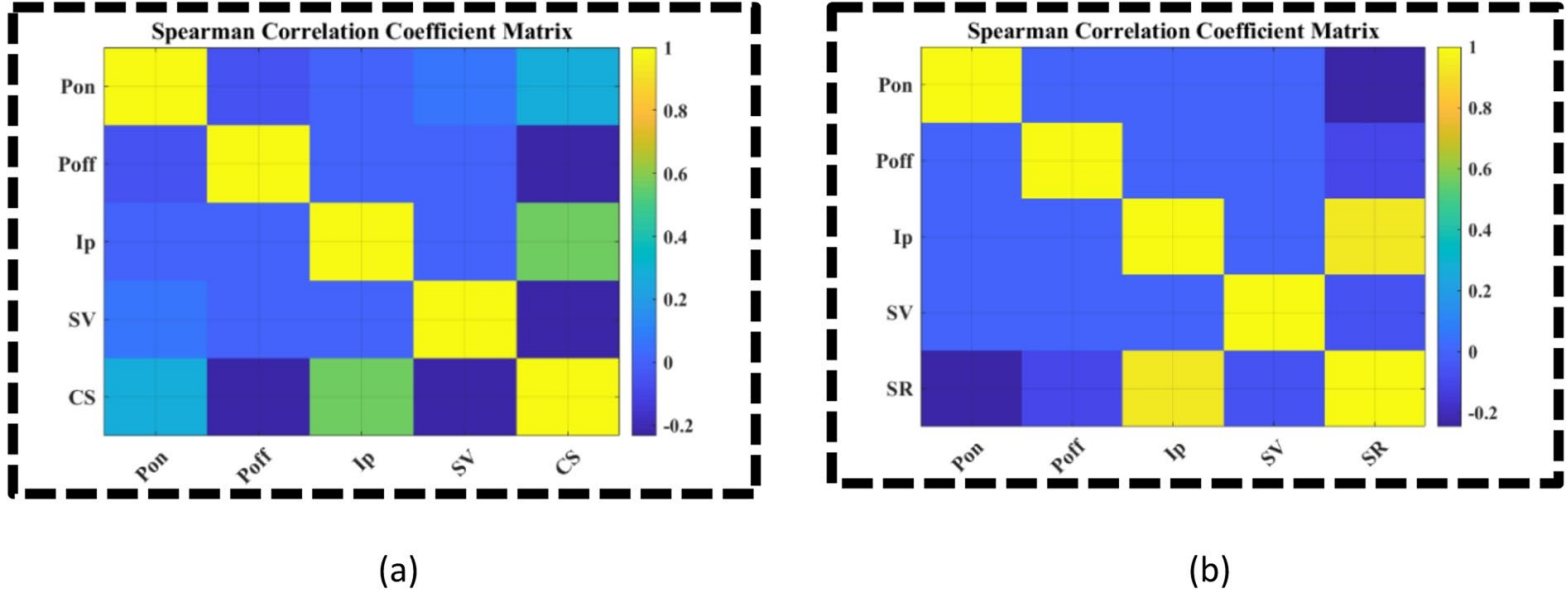
Spearman correlation coefficient matrix (**a**) Cutting speed (CS), and (**b**) Surface roughness (SR).

The sensitivity was conducted to gain a comprehensive understanding of the influence of the WEDM process parameters on the machining responses. It quantified not only the individual (main) effects of each parameter but also their interactive contributions, thereby providing deeper insight into the complex parameter–response relationships inherent to the WEDM process. Sensitivity analysis is a methodical technique employed to evaluate the impact of fluctuations in input parameters on the outputs of a model or system^34,35^. It assists in determining the most influential parameters, the strength of their effects on responses, and the presence of any interactions among them^36,37^. Sensitivity analysis improves the comprehension, dependability, and interpretability of predictive or experimental investigations by quantifying the impact of each input on the total variability of the outcome. This technique is extensively utilized in scientific and engineering domains to facilitate model validation, inform parameter selection, and enhance decision-making in optimization and process control. The outcomes of this analysis enhance the interpretability of the developed SVR models and offer a more robust foundation for the subsequent optimization using the evolutionary algorithm. The results of the sensitivity analyses for Cutting Speed (CS) and Surface Roughness (SR) are presented as Spearman correlation coefficient heatmaps in Fig. 3. As shown in Fig. 3a, Ip and Pon exhibit positive correlations with CS, while Poff and SV show negative correlations. Among these parameters, Ip is the most influential process variable for CS, with a correlation coefficient of approximately 0.6, followed by Pon with a coefficient of 0.4. In Fig. 3b, Ip is positively correlated with SR, whereas SV, Poff, and Pon are negatively correlated. The heatmap indicates that Ip is the most sensitive parameter affecting SR, with a correlation coefficient of approximately 0.9. In contrast, SV, Poff, and Pon have minimal impact on SR, with correlation coefficients of − 0.1, − 0.1, and − 0.2, respectively.

### Modelling of WEDM responses using optimized support vector regression (OSVR)

In this study, the correlation between the WEDM responses of Nickel Aluminium Bronze (NAB) alloy namely cutting speed (CS) and surface roughness (SR) and key input parameters such as servo voltage (SV), peak current (Ip), pulse-on time (Pon), and pulse-off time (Poff), is modeled using Optimized Support Vector Regression (OSVR). To enhance prediction accuracy, Bayesian Optimisation is employed to fine-tune the model’s hyperparameters and determine the optimal train-test split ratio. The resulting optimised models are then used for the prediction of CS and SR. Table 3 outlines the search space used for Bayesian hyperparameter optimisation, while Table 4 presents the final optimised hyperparameters obtained for the ML models corresponding to CS and SR.

**Fig. 4:**
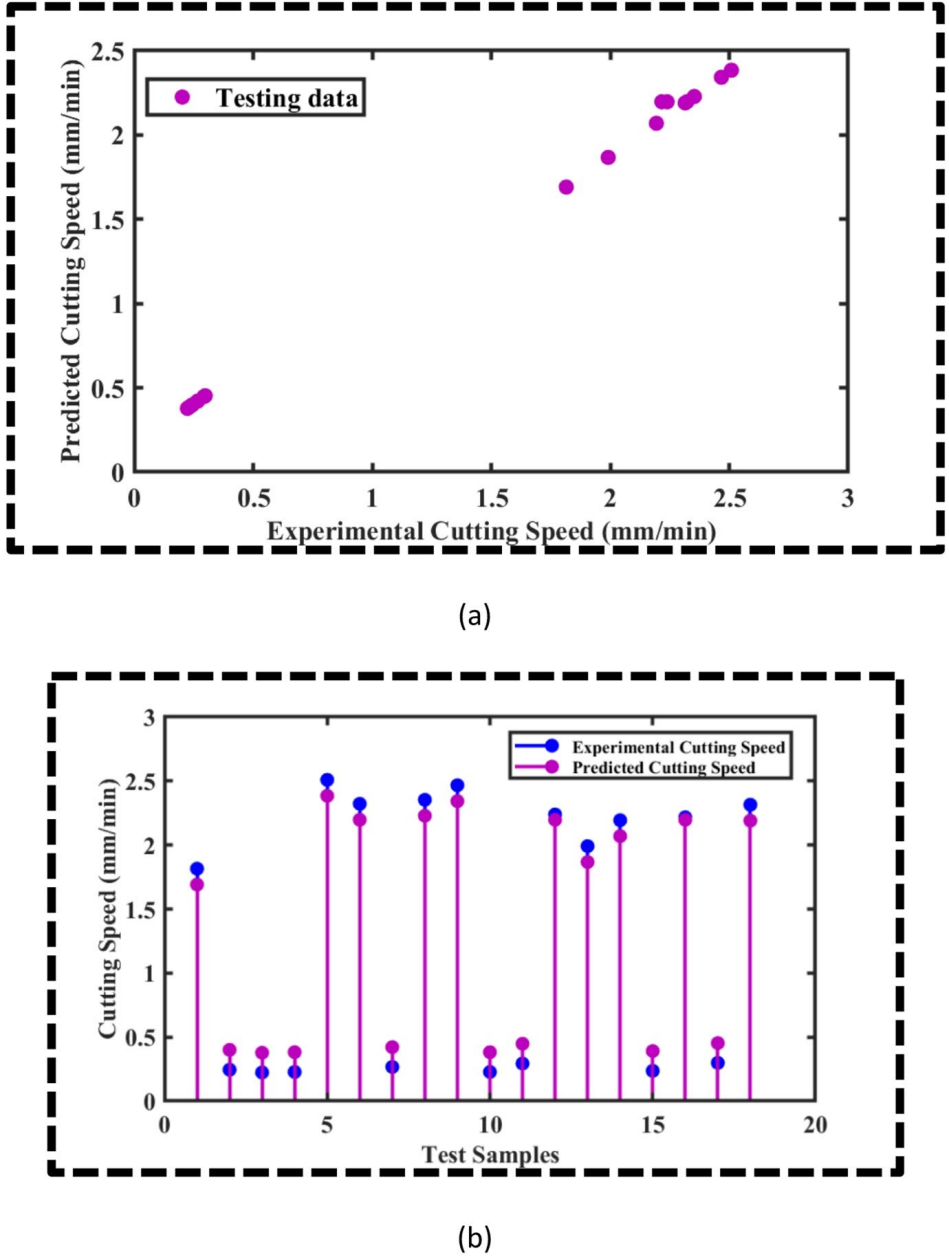
(**a**) Scatter plot between predicted CS and experimental CS, (**b**) Stem plot between predicted CS and experimental CS for the test samples.

**Fig. 5:**
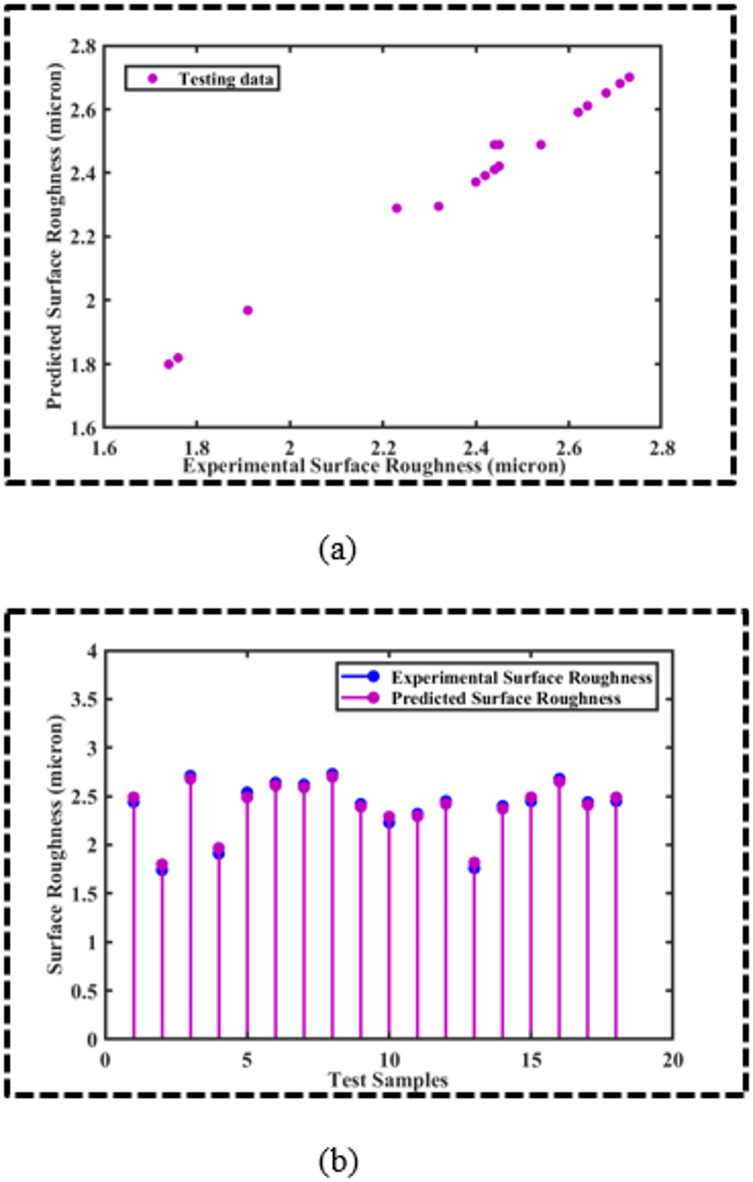
(**a**) Scatter plot between predicted SR and experimental SR, (**b**) Stem plot between predicted SR and experimental SR for the test samples.

The OSVR-based models for CS and SR yielded MSEs of 0.0027 and 0.0012, respectively, along with *R*^2^ values of 0.9970 and 0.9924 for CS and SR, which is impressive. However, to further corroborate the predictive accuracy of the ML models, scatter plots and stem plots are presented in Figs. 4 and 5 for the CS and SR, respectively. Figure 4a presents the scatter plot between the predicted cutting speed and the experimental cutting speed for the testing data. It is evident that the majority of the data points are aligned in such a way that they are hypothetically forming a straight line of slope 1, which implies that the OSVR model for CS is adequate to explain the data. Furthermore, a stem plot is provided in Fig. 4b, which endorses that the predicted cutting speed is in good agreement with the experimental cutting speed. Similarly, the scatter plot between the experimental and predicted surface roughness for the testing data is displayed in Fig. 5a. It is observed that the bulk of the data points are aligned to form a hypothetical straight line with a slope of 1, indicating that the model is sufficient to describe the SR data. Additionally, Fig. 5b presents a stem plot that confirms that the experimental SR and the predicted SR agree well. Thus, the OSVR model predicts the SR adequately.

**Table 3:** Hyperparameter search space considered for bayesian optimization.

C	0.001–1000
ε	0.0001–10
Ƴ	0.0001–10
Test size	0.1–0.9

**Table 4:** Post-optimized hyperparameters for ML models.

Hyperparameters	CS	SR
C	239.1661	9.3480
ε	0.0002	0.0001
Ƴ	2.8181	10
Test size	0.28	0.27

### Variation of cutting speed with process parametric variation

**Fig. 6:**
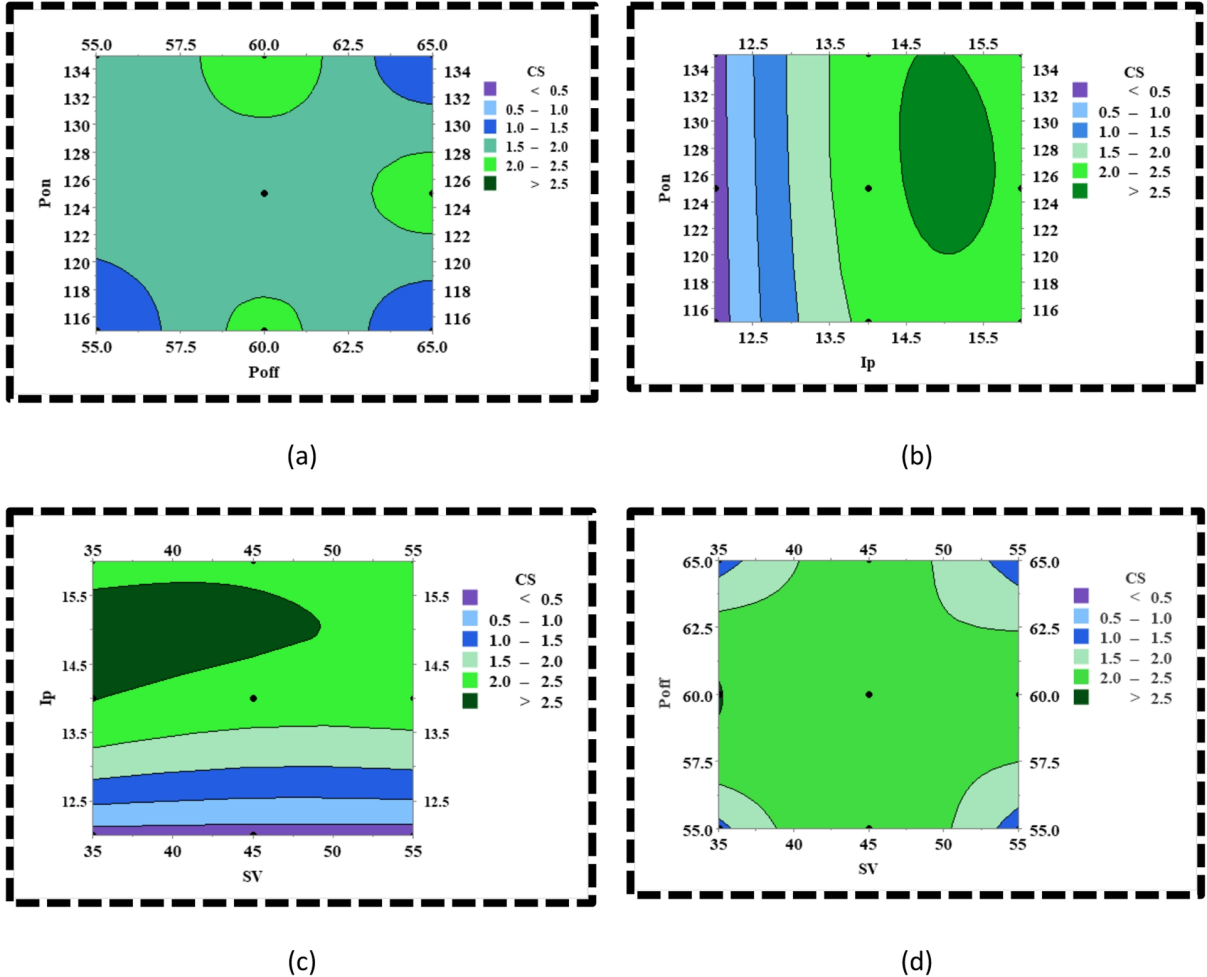
CS variation with variation in the process parameters (**a**) variation of CS with variation in Pon and Poff, (**b**) variation of CS with variation in Pon and Ip, (**c**) variation of CS with variation in Ip and SV, (**d**) variation of CS with variation in Poff and SV.

The contour plots in Fig. 6 demonstrate the variation of CS with the variation in the interaction between any pair of process parameters. The first contour plot describes the interaction between Pon and Poff and its corresponding effect on CS (Fig. 6a). It is evident that when Poff is at its minimum range, i.e., (55–56.5) μs, the CS increases with an increase in Pon but not substantially, as the dielectric flushing of the debris is poor relative to the quantity of material which is melted at higher Pon because of extreme heat generation. However, as the value of Poff is increased, the CS monotonically increases at a substantial rate as the material expulsion rate from the machining zone substantially improves with an increase in the dielectric flushing. In the case when Poff is set at its maximum range, the CS diminishes due to the large-scale heat sinking effect owing to the higher amount of dielectric flushing. The second contour plot refers to the interaction between Pon and Ip and its corresponding effect on CS (Fig. 6b). It is evident that at minimum range of Ip i.e., (12–14) A, there is least interaction between Pon and Ip because with increment in Pon along the vertical axis, the color band remains uniform which implies that the effect of Pon is relatively insignificant when Ip is set to its minimum range as it doesn’t induce any discernible change on the energy of the plasma channel. However, there is a plausible interaction effect between the Pon and Ip when the Ip is around (14–15) A as it is noted that when the Ip is around (14–15) A, the increment in Pon increases the CS. This is because, at the maximum value of Ip, the increase in Pon promotes the realization of Ip in the spark gap to its maximum value as it is set in the machine which in turn enhances the energy of the plasma channel tremendously resulting in extensive melting and vaporization of the material. The third contour plot refers to the role of interaction between SV and Ip on CS (Fig. 6c). It is observed that when the SV is set at its minimum range, that is (35–50) volt, the increase in Ip tends to increase the CS initially followed by a slight decrease in CS. This is attributed to the increase in the intensity and energy of the spark discharges as the interelectrode gap is minimum at minimum values of SV and the energy of the plasma channel goes on increasing with the increase in Ip. However, the slight decrement in CS is due to abnormal discharges because of debris accumulation in the spark gap. Again, when the SV is set at its maximum range, that is (50–55) volts, there is a monotonic increase in CS up to 2.5 mm/min with an increment in Ip. The increase of CS beyond 2.5 mm/min is undermined due to the reduction in the spark intensity owing to the wider interelectrode gap at maximum values of SV. The fourth contour plot refers to the role of interaction between SV and Poff on CS (Fig. 6d). As evident from the plot, it is distinctly visible that there is no notable interaction between SV and Poff on CS in the entire region of the contour plot except at the corners.

### Variation of surface roughness with process parametric variation

**Fig. 7:**
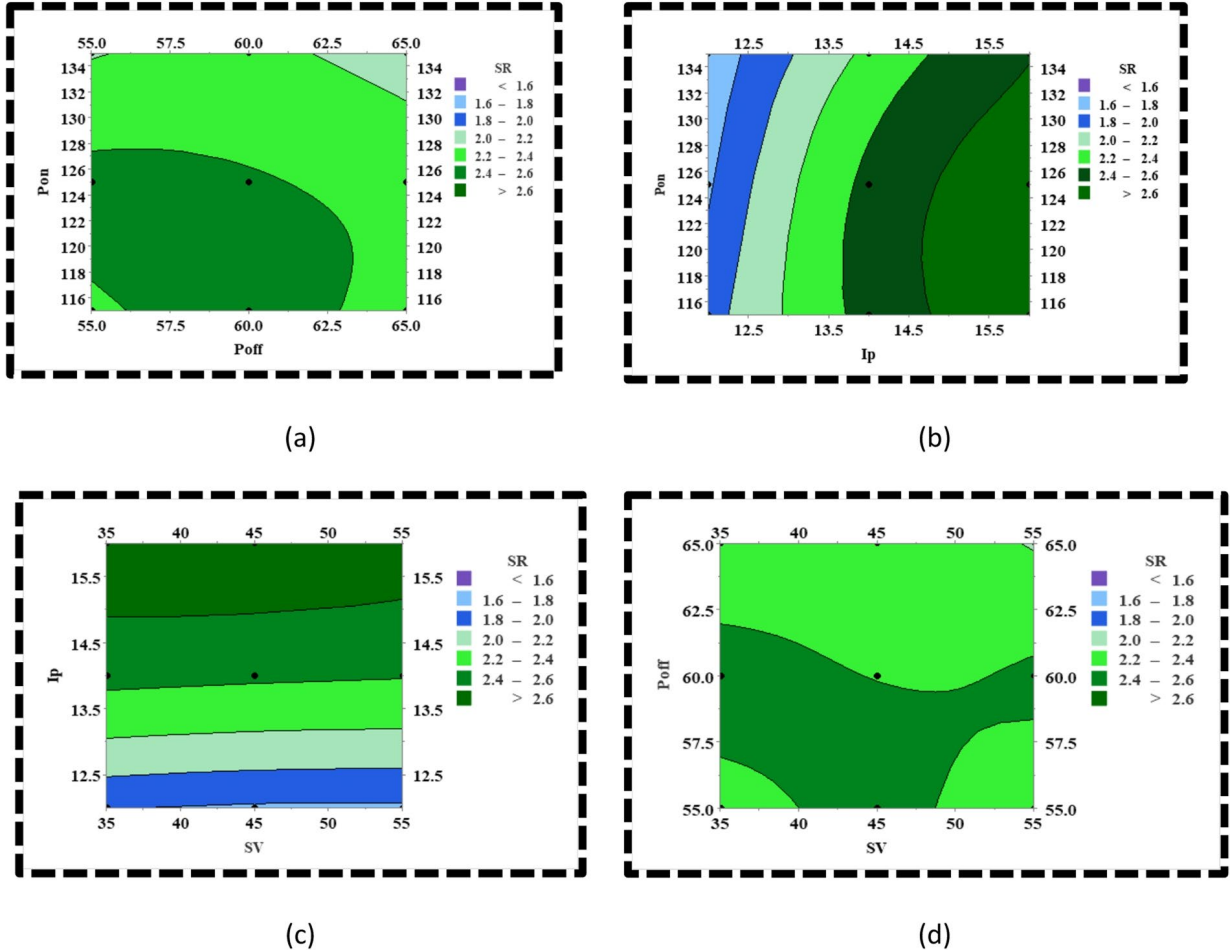
SR variation with variation in the process parameters (**a**) variation of SR with variation in Pon and Poff, (**b**) variation of SR with variation in Pon and Ip, (**c**) variation of SR with variation in Ip and SV, (**d**) variation of SR with variation in Poff and SV.

The contour plots in Fig. 7 elucidate the variation of SR with the variation in the interaction between any pair of process parameters. The first contour plot describes the interaction between Pon and Poff and its effect on SR (Fig. 7a). As evident from the plot, the SR shows an increasing trend when the Pon is increased from 0 to 127 µs and then starts to diminish as the Pon is increased from 127 to 135 µs. This trend of variation of SR happens when Poff is minimal. The increasing trend of SR can be attributed to the formation of large and deep-sized craters because of the increase in the Pon. On the contrary, the SR marginally decreases with a further increase in Pon because of the unflushed material, which gets redeposited on the machined surface due to minimum Poff. In other words, the redeposited material fills up the craters to some extent and creates smoother surfaces. However, the physical phenomena get altered when Poff increases. With the increase in Poff, as we move towards the right of the contour plot, the SR monotonically decreases with an increase in Pon along the vertical axis. This is due to the excessive heat-sinking effect at higher values of Poff, which results in minimal heat transfer to the base material even at maximum Pon, thereby triggering minimum melting and vaporisation of the material and forming narrow and shallow-sized craters. The second contour plot portrays the interaction effect between Pon and Ip and its effect on SR (Fig. 7b). It is evident from the plot that at any Ip, the SR tends to diminish with increasing Pon. This may be explained by the fact that the plasma channel tends to grow with increasing Pon, which reduces the energy density of the plasma channel and, as a consequence, undermines extensive melting and vaporisation of the material, resulting in small and shallow-sized craters. The third contour plot portrays the interaction effect between Ip and SV and its effect on SR (Fig. 7c). It is noticed that at any SV, the increase in Ip leads to enhancement of the surface roughness as corroborated through the change in the colour after regular intervals along the Y-axis, i.e., from light blue to dark green colour, which is indicative of minimum SR and maximum SR, respectively. The only difference that can be noticed along the X-axis is that a rapid change in the colour at minimum SV, whereas there is a gradual change in the colour at maximum SV. This implies that at minimum SV, the spark intensity is more owing to the narrower interelectrode gap, and the spark intensity is lower at maximum SV due to the wider interelectrode gap. The fourth contour plot, as depicted in Fig. 7d, portrays the interaction effect between Poff and SV and its effect on SR. At minimum SV that is within (35–40) volt, when the Poff is increased beyond 55–57 µs, the SR increases marginally as a relatively higher value of Poff promotes efficient flushing of debris from the spark gap thereby resulting in efficient spark discharges which are conducive to forming large and deep craters and increases the SR. However, on increasing the Poff further, there is a slight decline in the SR as it relatively increases the transference of thermal energy from the spark gap into the dielectric fluid, thereby diminishing the dimension of craters. Again, when the SV lies in the domain of (40–50) volts, there is a substantial flushing of dielectric fluid as the interelectrode gap is wider as compared to the earlier case. Therefore, there is no such region where the SR increases with increasing Poff. The reason that exists is that when Poff is increased beyond 60 µs, the SR reduces owing to a relatively larger heat transfer of spark-induced heat into the dielectric fluid, promoting the generation of relatively smaller-sized craters. Lastly, if the SV is increased beyond 50 volts, it is noted that the color of the entire region along the Y-axis i.e., the Poff axis is uniform with minimum SR except for a few regions with relatively larger SR. This is obvious as the maximum SV, regardless of Poff usually leads to a much wider interelectrode gap and thereby restricts the flow of heat to the substrate material tremendously triggering the generation of small-sized craters.

### Multi-objective optimization using IBEA-AOG

In Wire Electrical Discharge Machining (WEDM), attaining optimal results for both cutting speed (CS) and surface roughness (SR) simultaneously is challenging due to the complexity of the process. However, industrial applications generally favour a combination of high CS and low SR to enhance both productivity and product quality. As a result, this challenge is framed as a multi-objective optimization problem, where the aim is to maximize CS while minimizing SR at the same time, which is mathematically formulated as follows:

*Maximize* CS

*Minimize* SR

Constraints:

115 < P_on_ < 135,

55 < P_off_ < 65,

14 < I_p_ < 16

35 < SV < 55

**Fig. 8:**
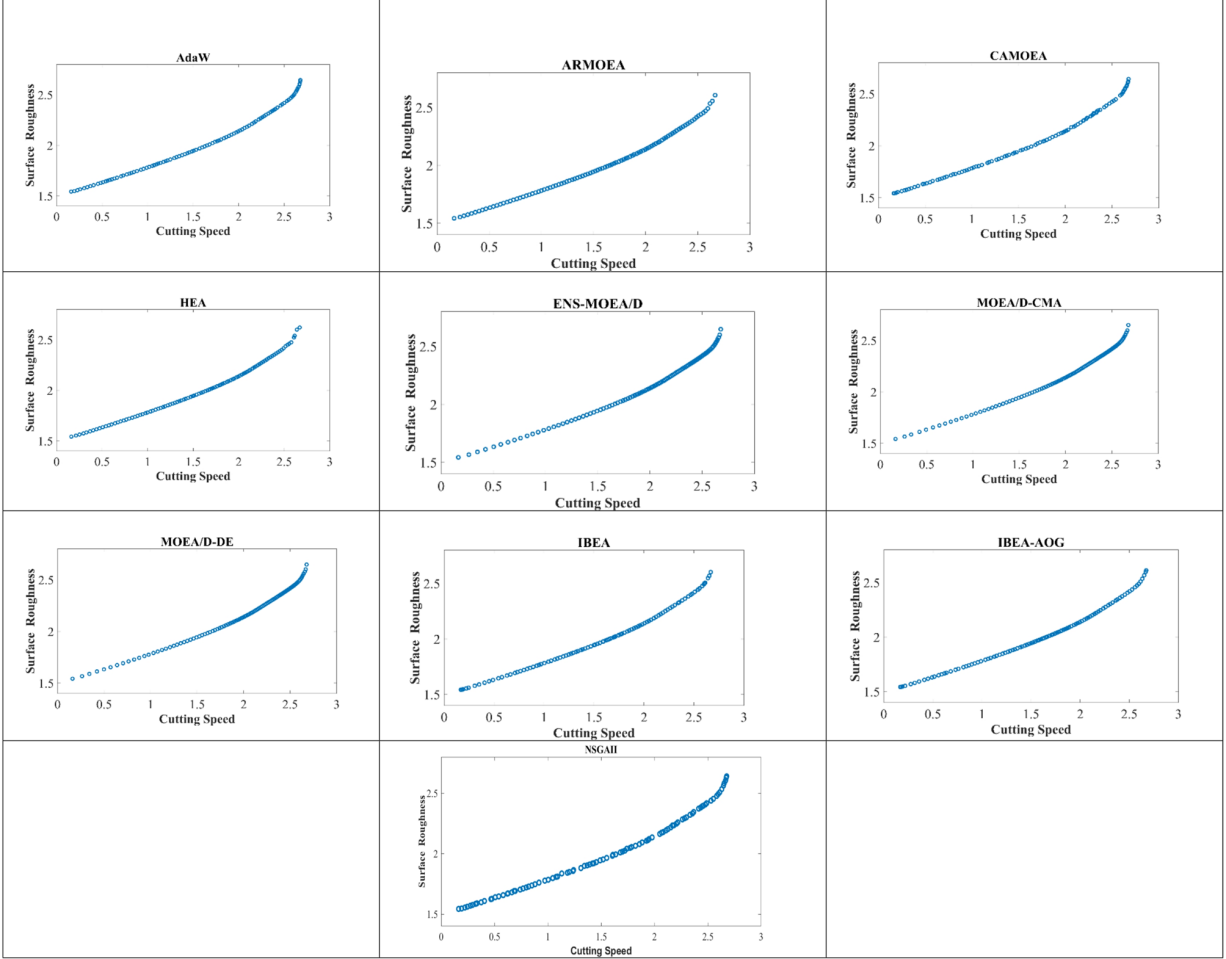
Pareto front plots of all the representative algorithms.

**Fig. 9:**
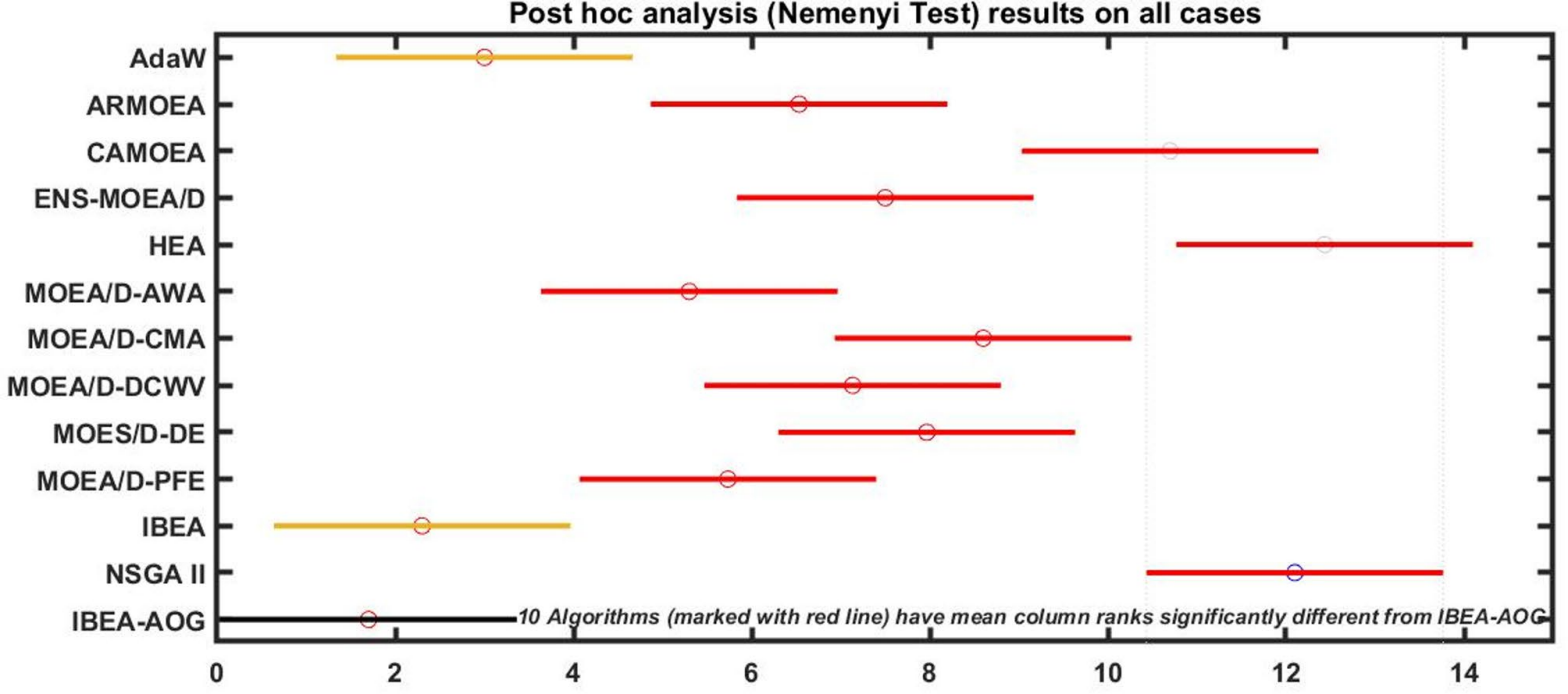
Post hoc analysis (Nemenyi test) results.

Initially, OSVR models for cutting speed (CS) and surface roughness (SR) are utilized as fitness functions in the proposed optimization approach, IBEA-AOG, to address the multi-objective optimization problem. The optimization approach, IBEA-AOG, is implemented using MATLAB, resulting in the extraction of a set of non-dominated Pareto-optimal solutions (Supplementary Table 1). The results obtained through IBEA-AOG are compared with twelve state-of-the-art algorithms (i.e., AdaW^38^, ARMOEA^39^, CAMOEA^40^, ENS-MOEA/D^41^, HEA^42^, MOEA/D-AWA^43^, MOEA/D-CMA^44^, MOEA/D-DCWV^45^, MOEA/D-DE^46^, MOEA/D-PFE^47^, NSGA II, and IBEA^48^. The hypervolumes (HVs) are calculated using the solutions obtained through each algorithm. Table 5 presents the mean and standard deviation of the HVs obtained through each representative algorithm over 30 independent runs. From the table, it can be observed that IBEA and IBEA-AOG outperform the results obtained through the rest of the state-of-the-art algorithms. IBEA-AOG achieved the best mean HVs when compared to the other algorithms. Analysing the Pareto Front plots of all the representative algorithms in Fig. 8, it is noted that the Pareto Front plot achieved by IBEA-AOG has relatively more uniformly dispersed Pareto solutions as compared to the Pareto solutions achieved by other competitive algorithms, which further corroborates the superiority of the IBEA-AOG over other algorithms. In addition, Friedman’s mean ranks and post hoc analysis (Nemenyi test) results are presented in Table 6; Fig. 9, respectively. IBEA-AOG obtains the best Friedman mean rank while AdaW and IBEA show competitive performance. In the post hoc analysis, it is confirmed that AdaW and IBEA obtain similar results, whereas the rest of the compared algorithms have obtained results that are significantly different from IBEA-AOG.

**Table 5:** HVs evaluated for competing algorithms.

Algorithm	AdaW	ARMOEA	CAMOEA	MOEA/D-AWA	MOEA/D-DCWV	MOEA/D-PFE	HEA	ENS-MOEA/D	MOEA/D-CMA	MOEA/D-DE	IBEA	NSGA-II	IBEA-AOG
HV	6.9504e−1 (1.52e−4) –	6.9449e−1 (4.69e−4) –	6.9371e−1 (2.61e−4) –	6.9468e−1 (1.57e−4) –	6.9388e−1 (2.18e−3) –	6.9457e−1 (5.90e−4) –	6.9168e−1 (1.69e−3) –	6.9442e−1 (9.29e−5) –	6.9430e−1 (1.71e−4) –	6.9437e−1 (1.15e−4) –	6.9512e−1 (2.12e−4) =	6.9291e−1 (2.71e−4) –	6.9519e−1 (1.29e−4)
**+/**−**/=**	0/1/0	0/1/0	0/1/0	0/1/0	0/1/0	0/1/0	0/1/0	0/1/0	0/1/0	0/1/0	0/0/1	0/0/1	

**Table 6:** Friedman mean ranks.

Algorithm	AdaW	ARMOEA	CAMOEA	ENS-MOEA/D	HEA	MOEA/D-AWA	MOEA/D-CMA	MOEA/D-DCWV	MOEA/D-DE	MOEA/D-PFE	IBEA	NSGA II	IBEA-AOG
Friedman ranks	3.00	6.53	10.70	7.50	11.63	5.30	8.60	7.03	7.97	5.73	2.30	12.1	1.70

### Evaluation of surface topography

**Fig. 10:**
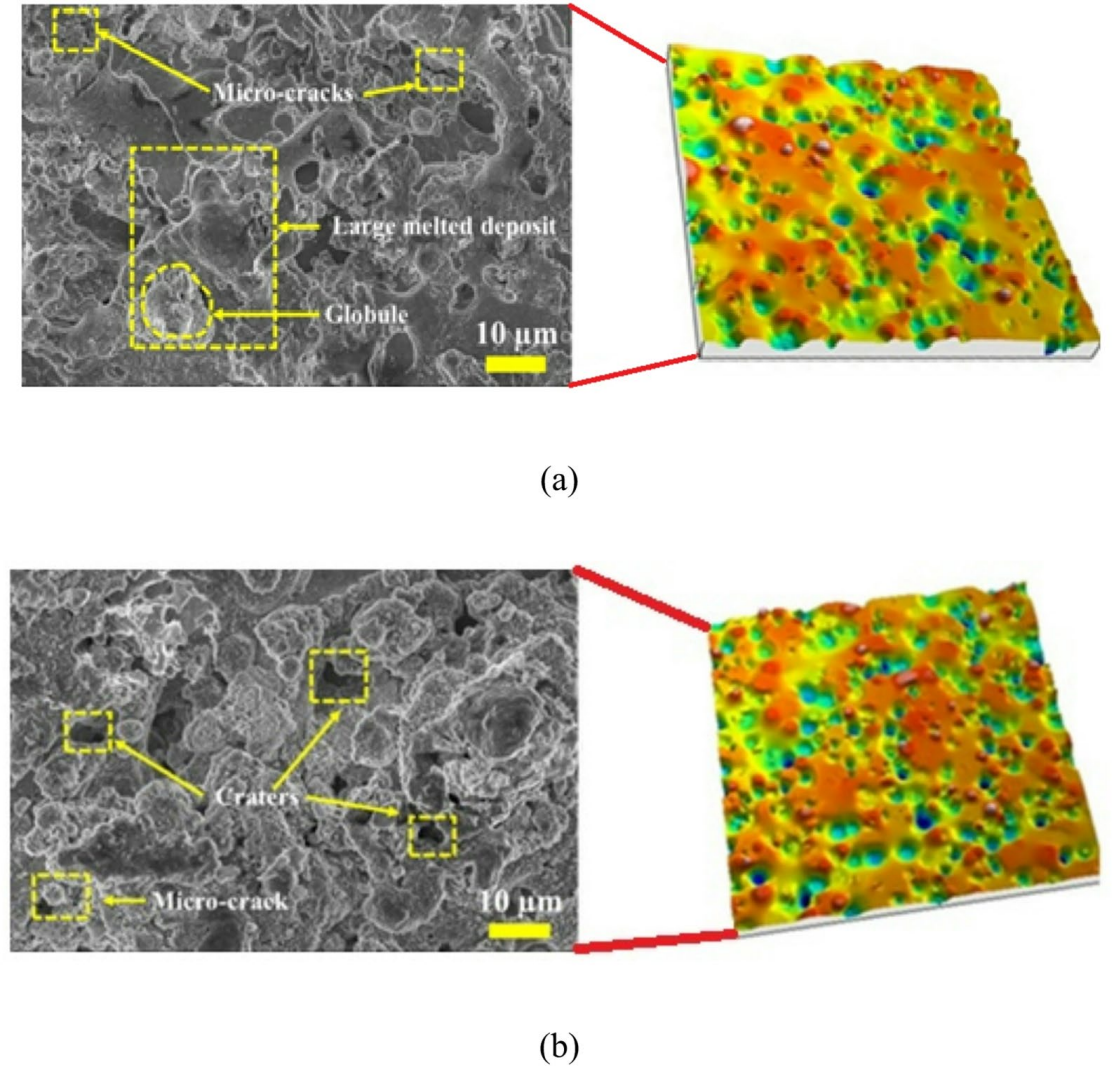
FESEM micrograph with non-contact optical profilometer’s image at (**a**) high discharge energy setting (Pon = 135 µs, Poff = 55 µs, Ip = 16 A, and SV = 35 vol) and (**b**) low discharge energy setting (Pon = 115 µs, Poff = 65 µs, Ip = 12 A, and SV = 55 V).

In the present research, the surface topography of the machined surfaces is analysed through FESEM and non-contact optical profilometer images for varying parametric settings. These settings correspond to high discharge energy settings, namely Pon = 135 µs, Poff = 55 µs, Ip = 16 A, and SV = 35 volts (Fig. 10a), and low discharge energy settings, namely Pon = 115 µs, Poff = 65 µs, Ip = 12 A, and SV = 55 volts (Fig. 10b). Analyses indicate that the surface generated at high discharge energy settings comprises globules and significant amounts of large melted deposits, corroborating that the molten material produced during machining redeposited due to inadequate dielectric flushing, resulting in overlapping craters. In contrast, the surface generated at low discharge energy settings consists of a loosely bonded recast layer and craters due to the generation of less molten material, which is effectively flushed away through the dielectric flow. In addition, microcracks are visible on the surfaces for both low-energy and high-energy settings. The quantity of microcracks is relatively higher on surfaces at high energy settings, attributable to large thermal gradients, but lower for surfaces at low energy settings due to relatively smaller thermal gradients.

## Conclusions

The present study delivers substantial scientific and industrial contributions through the development of a hybrid OSVR–IBEA-AOG framework for accurate prediction and multi-objective optimisation in WEDM of NAB alloy. Scientifically, an Optimized Support Vector Regression model was established to capture the nonlinear behaviour of spark erosion with exceptionally high predictive accuracy for both cutting speed and surface roughness. The Indicator-Based Evolutionary Algorithm was further enhanced through an adaptive offspring-generation mechanism (IBEA-AOG), resulting in superior convergence and solution diversity compared to eleven benchmark algorithms, as validated through Friedman and Nemenyi statistical tests. The integrated prediction–optimisation workflow, supported by Spearman correlation analysis along with FESEM and optical profilometry examinations, advances the understanding of parameter influence, crater evolution, recast layer formation, and microcracking mechanisms under varying discharge energies.

From an industrial perspective, the framework offers a reliable tool for predicting machining performance, reducing experimental time and cost, and delivering 100 Pareto-optimal solutions to support informed decisions regarding productivity–quality trade-offs. The morphological insights further assist manufacturers in minimising surface defects and enhancing the integrity of NAB alloy components, while the identification of low-energy parameter settings supports more sustainable machining practices. The proposed framework is also generic and transferable, facilitating broader industrial adoption of AI-enabled optimisation for advanced manufacturing processes. The key conclusions of the study are summarised as follows:

- The OSVR models demonstrated high prediction accuracy, achieving MSE values of 0.0027 and 0.0012, and *R*^2^ values of 0.9970 and 0.9924 for CS and SR, respectively.
- The IBEA-AOG achieved the best mean HVs when compared to the other algorithms. The Pareto Front plot achieved by IBEA-AOG is relatively more uniformly dispersed as compared to the Pareto front plots achieved by other competitive algorithms.
- The CS increased with higher Poff due to improved debris flushing, but excessive Poff led to heat loss and reduced CS. Significant interaction between Pon and Ip was observed only at higher Ip levels, as increased plasma energy enhanced material removal.
- The interaction between SV and Ip on CS revealed that at low SV (35–50 V), CS initially increases with Ip due to higher spark energy, but then slightly decreases due to abnormal discharges from debris accumulation. At high SV (50–55 V), CS increases monotonically with Ip up to 2.5 mm/min, beyond which it stabilises due to reduced spark intensity caused by a wider interelectrode gap.
- SR initially increased with Pon at low Poff resulting in deeper craters, then decreased due to redeposition of unflushed debris. However, at higher Poff, SR consistently decreased with Pon due to heat dissipation. Across Ip–Pon interaction, SR decreased with Pon as plasma channel expansion reduced the energy density.
- In the Ip–SV interaction, SR increases with Ip at all SV levels, more sharply at lower SV due to higher spark intensity. For the Poff–SV interaction, SR slightly increases at low SV and moderate Poff due to better flushing, but generally decreases with increasing Poff and SV as wider gaps and greater heat transfer to the dielectric limit crater size.
- The surface topography revealed that high discharge energy settings produce surfaces with large molten deposits, overlapping craters, and more microcracks due to poor flushing and high thermal gradients. In contrast, low discharge energy settings result in smoother surfaces with fewer microcracks, a loosely bonded recast layer, and well-flushed, shallow craters due to reduced molten material and lower thermal gradients.

## Supplementary Information

Below is the link to the electronic supplementary material.

Supplementary Material 1

## Data Availability

The datasets used and/or analysed during the current study available from the corresponding author on reasonable request.
