# Supplementary material for: Predictive modelling and optimization of WEDM of nickel aluminium bronze alloy using optimised support vector regression and evolutionary algorithm
Source: Sci Rep. 2025 Dec 30;16:3982. doi: 10.1038/s41598-025-34151-8 (PMC12855905; doi:10.1038/s41598-025-34151-8)
Supplement: Supplementary file 1 — Supplementary Material 1 [file 41598_2025_34151_MOESM1_ESM.docx]

**Supplementary Table 1** Non-dominated solutions retrieved through IBEA-AOG

| Pon (microsec) | Poff (microsec) | Ip (A) | SV (volt) | CS (m/min) | SR (micron) |
| --- | --- | --- | --- | --- | --- |
| 135 | 64.99972 | 13.35214 | 51.5504 | 1.722341 | 2.025446 |
| 134.9998 | 64.99993 | 13.17247 | 49.92028 | 1.564033 | 1.967567 |
| 135 | 65 | 13.06921 | 51.31617 | 1.478997 | 1.937656 |
| 134.9994 | 65 | 13.43741 | 51.0995 | 1.784447 | 2.049296 |
| 135 | 65 | 13.33773 | 51.12192 | 1.708433 | 2.020129 |
| 135 | 65 | 13.81234 | 49.18724 | 2.017543 | 2.147924 |
| 134.9974 | 64.70646 | 14.17417 | 41.55028 | 2.204684 | 2.246477 |
| 135 | 65 | 13.2302 | 50.80926 | 1.618661 | 1.98699 |
| 135 | 65 | 13.09263 | 51.38529 | 1.501055 | 1.945296 |
| 135 | 65 | 13.41664 | 50.24578 | 1.764539 | 2.041511 |
| 134.9997 | 63.77524 | 14.1967 | 39.51305 | 2.256995 | 2.27618 |
| 134.9992 | 65 | 13.14555 | 50.21491 | 1.541778 | 1.959652 |
| 135 | 65 | 13.24497 | 50.78455 | 1.630962 | 1.991455 |
| 134.9995 | 64.99987 | 13.21017 | 50.69114 | 1.600932 | 1.980608 |
| 135 | 65 | 12.84825 | 51.87491 | 1.265404 | 1.866128 |
| 135 | 65 | 13.04573 | 51.31617 | 1.457055 | 1.930111 |
| 135 | 65 | 13.73316 | 49.95891 | 1.975129 | 2.128565 |
| 135 | 65 | 12.49413 | 52.15787 | 0.866594 | 1.741287 |
| 134.9994 | 64.99496 | 14.03549 | 46.19049 | 2.124488 | 2.201399 |
| 135 | 65 | 13.62637 | 49.95981 | 1.909432 | 2.099986 |
| 134.9999 | 65 | 13.50474 | 50.24578 | 1.828685 | 2.066751 |
| 135 | 65 | 12.94579 | 51.29248 | 1.360314 | 1.897417 |
| 135 | 65 | 12.89996 | 51.31764 | 1.314467 | 1.882219 |
| 134.9994 | 64.99964 | 14.08482 | 45.81584 | 2.145712 | 2.212995 |
| 135 | 64.55228 | 14.12911 | 43.05517 | 2.185402 | 2.235407 |
| 134.9998 | 65 | 13.70119 | 49.98884 | 1.9562 | 2.120168 |
| 135 | 65 | 14.12515 | 44.90946 | 2.163389 | 2.222941 |
| 135 | 65 | 13.11716 | 50.87199 | 1.520086 | 1.951939 |
| 129.4007 | 55 | 14.91938 | 35 | 2.671434 | 2.612095 |
| 135 | 64.99953 | 13.47395 | 50.20554 | 1.806544 | 2.057938 |
| 135 | 65 | 12 | 48.51813 | 0.167881 | 1.543107 |
| 135 | 65 | 13.28805 | 50.24578 | 1.663694 | 2.003481 |
| 135 | 61.76707 | 14.32016 | 35 | 2.404474 | 2.361011 |
| 135 | 64.99965 | 13.9365 | 49.28648 | 2.081914 | 2.179201 |
| 135 | 65 | 12.21561 | 53.14957 | 0.515467 | 1.637746 |
| 135 | 65 | 13.53227 | 50.14045 | 1.847426 | 2.074307 |
| 135 | 65 | 12.31275 | 52.37597 | 0.638068 | 1.673375 |
| 135 | 65 | 13.26226 | 51.31617 | 1.648552 | 1.9979 |
| 134.9999 | 65 | 13.02745 | 50.65418 | 1.435489 | 1.92281 |
| 135 | 65 | 13.89978 | 49.09367 | 2.063273 | 2.169859 |
| 135 | 55.39512 | 14.5944 | 35 | 2.578607 | 2.473832 |
| 135 | 65 | 13.85827 | 49.08655 | 2.041914 | 2.15946 |
| 135 | 63.03497 | 14.21022 | 35 | 2.329122 | 2.317458 |
| 135 | 65 | 13.77039 | 49.80066 | 1.996077 | 2.138029 |
| 135 | 65 | 12.79443 | 52.15742 | 1.210873 | 1.848479 |
| 135 | 65 | 12.54339 | 52.1215 | 0.925958 | 1.759306 |
| 135 | 65 | 12.51939 | 51.91115 | 0.895213 | 1.749978 |
| 135 | 65 | 13.66416 | 50.51515 | 1.935698 | 2.111264 |
| 135 | 65 | 12.16888 | 53.07967 | 0.450326 | 1.619032 |
| 131.9477 | 55 | 14.84983 | 35 | 2.651863 | 2.567487 |
| 135 | 65 | 13.19014 | 50.14045 | 1.580603 | 1.973397 |
| 135 | 61.8697 | 14.25129 | 35 | 2.377664 | 2.345155 |
| 134.9998 | 63.78271 | 14.14947 | 39.8329 | 2.23603 | 2.264281 |
| 134.9987 | 64.99682 | 12.99545 | 51.08638 | 1.407667 | 1.913378 |
| 135 | 65 | 12.44754 | 52.39072 | 0.811111 | 1.724583 |
| 135 | 60.36848 | 14.2862 | 35 | 2.426048 | 2.373418 |
| 135 | 65 | 12.71626 | 52.15787 | 1.125751 | 1.821318 |
| 135 | 65 | 12.56623 | 52.66975 | 0.958103 | 1.769149 |
| 135 | 62.64955 | 14.2104 | 38.30218 | 2.305835 | 2.304087 |
| 135 | 56.35294 | 14.52969 | 35 | 2.55765 | 2.457614 |
| 135 | 65 | 12.46273 | 53.3292 | 0.839037 | 1.733064 |
| 134.9999 | 65 | 13.31381 | 50.24578 | 1.684582 | 2.011214 |
| 135 | 65 | 12 | 49.54856 | 0.175255 | 1.54375 |
| 135 | 65 | 12.06861 | 53.64723 | 0.314566 | 1.58049 |
| 135 | 62.3695 | 14.24993 | 35 | 2.363521 | 2.33703 |
| 135 | 65 | 12.10675 | 52.71148 | 0.359044 | 1.593075 |
| 135 | 64.9792 | 13.9786 | 48.84428 | 2.101455 | 2.189264 |
| 135 | 55.00065 | 14.67447 | 35 | 2.595862 | 2.489023 |
| 135 | 57.6626 | 14.46405 | 35 | 2.527375 | 2.436503 |
| 135 | 65 | 12.59814 | 52.01797 | 0.989933 | 1.778925 |
| 135 | 65 | 12 | 51.29909 | 0.190071 | 1.54625 |
| 135 | 65 | 12.97538 | 51.32256 | 1.389649 | 1.907236 |
| 135 | 65 | 12.34101 | 53.07967 | 0.681998 | 1.686258 |
| 135 | 65 | 12 | 53.825 | 0.216525 | 1.552978 |
| 135 | 65 | 12.76553 | 52.12949 | 1.17954 | 1.838423 |
| 130.5294 | 55 | 14.91938 | 35 | 2.666131 | 2.597645 |
| 135 | 55 | 14.80125 | 35 | 2.613827 | 2.509643 |
| 135 | 65 | 12.66351 | 51.8877 | 1.064269 | 1.801997 |
| 135 | 65 | 12.82269 | 51.44684 | 1.235365 | 1.856388 |
| 135 | 65 | 12.86742 | 52.10207 | 1.287056 | 1.873218 |
| 135 | 63.45976 | 14.23173 | 38.66023 | 2.286669 | 2.293236 |
| 135 | 65 | 12.20016 | 52.90115 | 0.491706 | 1.630902 |
| 135 | 58.1049 | 14.40213 | 35 | 2.503754 | 2.420946 |
| 135 | 65 | 12.0442 | 53.20655 | 0.274304 | 1.569135 |
| 135 | 65 | 12.73586 | 52.15726 | 1.147388 | 1.828176 |
| 135 | 58.78746 | 14.36326 | 35.01669 | 2.481276 | 2.406798 |
| 133.5108 | 55 | 14.80365 | 35 | 2.632462 | 2.535848 |
| 135 | 65 | 12.24579 | 53.32847 | 0.558338 | 1.650157 |
| 135 | 65 | 12.41167 | 51.65637 | 0.75945 | 1.709289 |
| 135 | 65 | 12.29587 | 52.73436 | 0.619247 | 1.667848 |
| 135 | 65 | 13.58854 | 50.32907 | 1.88633 | 2.090296 |
| 135 | 65 | 12.27609 | 52.36598 | 0.589327 | 1.659166 |
| 135 | 65 | 13.558 | 50.65418 | 1.867414 | 2.082492 |
| 135 | 59.54636 | 14.32042 | 35 | 2.454272 | 2.390273 |
| 135 | 65 | 12.91907 | 51.31764 | 1.333788 | 1.888602 |
| 135 | 65 | 12.14223 | 53.03219 | 0.412578 | 1.608253 |
| 135 | 65 | 12.68362 | 51.91115 | 1.087227 | 1.809184 |
| 134.9997 | 65 | 13.3859 | 50.82924 | 1.744237 | 2.033706 |
| 135 | 65 | 12.63753 | 51.89699 | 1.034633 | 1.792765 |
| 135 | 65 | 12.38015 | 52.89211 | 0.730667 | 1.700619 |
